# A Continuous Oral Regimen of High-Dose Cromolyn Sodium Is Effective for Some Myalgic Encephalomyelitis/Chronic Fatigue Syndrome (ME/CFS) Patients With Mast Cell Activation Syndrome

**DOI:** 10.7759/cureus.102064

**Published:** 2026-01-22

**Authors:** Maritsa E Christoforou, Linda C van Campen, Frans C Visser, Carlton K Lee, Samantha L Lemmon, Peter C Rowe, Alba M Azola

**Affiliations:** 1 Pediatrics, Johns Hopkins University School of Medicine, Baltimore, USA; 2 Cardiology, Stichting CardioZorg, Hoofddorp, NLD

**Keywords:** disodium cromoglycate, long covid, mast cell activation syndrome, mastocytosis, myalgic encephalomyelitis/chronic fatigue syndrome, oral cromolyn sodium, orthostatic intolerance

## Abstract

Our clinical experience in the last four years using oral cromolyn in patients with mast cell activation syndrome (MCAS) suggests that a continuous oral regimen of high-dose cromolyn may enhance compliance with the medication. The five patients described in this retrospective case series were given instructions to take oral cromolyn using a continuous dosing regimen, placing the entire day’s dose in an opaque bottle that is then filled with water, and sipping the solution throughout the day. If a conventional maximum dose of eight vials daily (800 mg) was tolerated but ineffective after a week, the patients were instructed to increase to 1600-2400 mg daily until reaching an optimal effect. We report that a cromolyn dose of 1600-2400 mg daily, administered using the continuous oral dosing regimen during the day, was effective in controlling signs and symptoms of mast cell activation. All five patients benefitted from a dose of cromolyn that is higher than usual and customary recommendations, but within the safety guidelines of the original Food and Drug Administration (FDA) application. The continuous oral regimen has some theoretical advantages over four discrete doses per day, though further study is needed.

## Introduction

Cromolyn sodium was first synthesized in 1965, after historical information emerged about the use of the Ammi visnaga plant in ancient Egyptian civilizations to minimize respiratory symptoms [1]. The active ingredient in the plant was the chromone, khellin, which binds to and stabilizes mast cell membranes, preventing the release of histamine and leukotrienes [1,2]. Cromolyn, in an inhaled form (known by the brand name of Intal®), has been used for many years for the prevention of asthma symptoms. Similarly, the nasal spray form, Nasalcrom®, is used in the treatment of allergic rhinitis, and the eye drop form, Opticrom®, is used for allergic conjunctivitis [3]. The oral form of cromolyn (Gastrocrom®) has primarily been used in the past to treat food allergies but has also shown efficacy in treating mast cell disorders [4,5]. Cromolyn is commercially available in some countries in tablet and capsule forms and in many countries is available in a compounded form. 

Mast cells are crucial in the immune response to harmful bacteria, parasites, viruses, and allergens, as they release multiple mediators that initiate a systemic response [6]. Mastocytosis is a rare disease that results from an overproduction of mast cells, regardless of the necessity of an immune response [7]. Mast cell activation syndrome (MCAS) is a more recently recognized condition characterized by persistent, but episodic symptoms similar to allergic inflammation [8,9]. In contrast to allergic reactions, MCAS can have multiple potential triggers in the absence of IgE mediation. Although there is disagreement about the diagnostic criteria for MCAS, all definitions require a multisystemic reaction, with symptoms in at least two of the following areas: skin (hives, rashes, swelling), respiratory (asthma, nasal congestion), cardiovascular (low blood pressure, faintness, palpitations), and digestive (esophageal dysfunction, vomiting, nausea, constipation/diarrhea) systems [10,11].

 Oral cromolyn is used to treat both systemic mastocytosis and MCAS. In the United States, it is primarily available as a liquid preparation in a concentration of 100 mg/5mL. In the FDA label, the recommended initial dose for adolescents and adults is 200 mg four times a day, administered 30 minutes before each of the three main meals and again at bedtime [12]. This dosage timing was intended primarily for those being treated for food allergy [13], but in general, patient adherence to any four-dose daily regimen tends to be low [14]. This case series emphasizes that an alternative regimen may enhance effectiveness, namely, placing all of the cromolyn doses for the day in an opaque water bottle, filling the bottle with water, and sipping from this solution intermittently throughout the day. In these individuals, exceeding the 800 mg daily dose was both safe and necessary to achieve an optimal therapeutic effect.

## Case presentation

Cases

Participants

All five patients met the Consensus-2 definition of MCAS. The Consensus-2 criteria can be satisfied by the existence of a unique constellation of multisystem clinical complaints attributable to pathologically increased mast cell activity (in the absence of any other disease better accounting for the patient’s problems), and a symptomatic response to inhibitors of mast cell activation or mast cell mediator production or action. Evidence of above-normal levels of mast cell mediators (including tryptase, histamine, or its metabolites such as N-methylhistamine, heparin, chromogranin A, prostaglandin D2, its metabolite 11-β-PGF2α, or leukotriene E4) is supportive of the diagnosis but not necessary if the above criteria are met [11]. Therefore, extensive testing for mast cell mediators was not performed routinely. Reported reference range values are specific to the individual laboratories where blood samples were processed. 

Continuous Oral Dosing Regimen for Cromolyn

The following instructions are given to patients in our clinic for initiating cromolyn treatment: "The starting dose for the first week is four 100 mg/5 mL vials daily (400 mg = 20 mL). After a week, increase the dose to eight vials per day (800 mg = 40 mL). In week 3, if there has been inadequate improvement, increase to 12 vials daily (1200 mg = 60 mL). In week 4, if there has been inadequate improvement, increase to 16 vials daily (1600 mg = 80 mL). If you increase the dosage and see no benefits within a week, return to the dosing where improvement was first noticed.” We recommend that our patients add the entire daily dose of cromolyn to their metal water bottle, then fill the bottle with non-alkaline water. Sipping from this throughout the day achieves a slow and more continuous distribution of the dose. Cromolyn should only be mixed with water, not with juice, milk, food, or anything else. We instruct patients to keep the vials out of direct sunlight and not take the ampule of liquid out of the foil package until they are ready to use it. We advise them to store the unopened ampules of liquid at room temperature, away from heat and direct light. There is not a fixed amount of water to add to the cromolyn, but larger volumes of water help reduce any adverse tastes the medication may have as well [12]. During increases in the oral cromolyn dose, other medications remained unchanged.

Wellness Score

Clinical outcomes were assessed at the discretion of each clinician using the Wellness score, which asked: “On a scale of 0 to 100, with 0 being 'dying' and 100 being 'the best you can imagine a person to feel', how would you rate yourself, on average, over the last month?” The wellness score is a reliable unidimensional health-related quality of life measure that correlates well with other widely used self-rating instruments [15] and is sensitive to clinical improvement in ME/CFS patients [15-17].

Case series

Pertinent summary data for the five cases are presented in Table 1. 

**Table 1:** Summary of the demographic and clinical data of the five cases. Abbreviations: M, male; F, female; T, tryptase; H, histamine; CGA, chromogranin A; qD, once every day; qAM, once every morning; BID, twice daily; PEM, post-exertional malaise; n/a, not available. Bold laboratory numbers indicate elevated levels. Reference range values for each patient are presented in the text.

Case	Age/sex	Tests	Medications pre-cromolyn	Wellness pre-cromolyn	Cromolyn dose (mg/day)	Months of follow-up	Wellness at the latest follow-up	Symptom changes/comments
1	16/M	T 1.3, 1,9; H n/a; CGA 34	None	20/100	2000	30	70/100	Improved energy, better tolerance of physical activity and orthostatic stress, and less light-headedness
2	37/F	T 3.0; H 0.26; CGA 32.7	Cetirizine 10 mg qD; NasalCrom spray; famotidine 40 mg BID; opticrom drops	26/100	1600	42	61/100	Improved facial flushing, rashes, periorbital/facial swelling, and energy. Less PEM, Opticrom helped conjunctival erythema and gritty sensation in eyes, but the greatest changes were from oral cromolyn
3	25/M	T 3.0, 2.8; H 12.6; CGA 68	Montelukast 10 mg qD; loratadine 10 mg q AM; famotidine 40 mg BID	30/100	1600	12	70-80/100	Improved facial flushing, hives, pruritus, energy, and activity
4	19/F	T 1.8; H < 1.5; CGA 88	Levocetirizine 5 mg qD; hydroxyzine 10 mg BID; famotidine 40 mg BID; NasalCrom spray	60/100	1600	24	86/100	Improved urticaria, rashes, headaches, lightheadedness; decreased cardiac awareness
5	41/F	T 4.8; H 0.29; CGA <20	Loratadine 20 mg BID; famotidine 20 mg BID; montelukast 10 mg qD; fluticasone nasal spray; NasalCrom spray	n/a	2400	22	n/a	Improved abdominal cramps, diarrhea, bloating, fatigue, brain fog, fewer naps, and higher daily step count

Case 1

**Figure 1:**
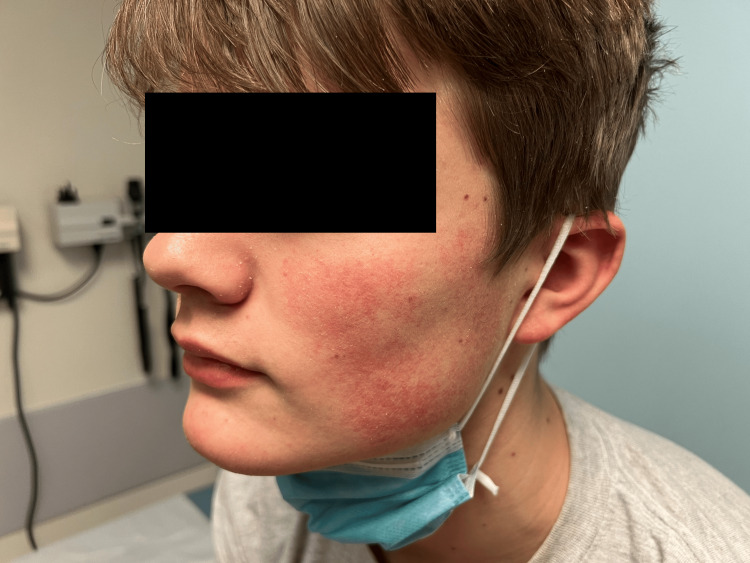
Facial erythema in Case 1. Note the extensive erythema affecting the cheeks and lower face (present bilaterally). A similar degree of erythema was also present on the triceps area of each arm, attributed to keratosis pilaris.

A 16-year-old male was evaluated in the Johns Hopkins Chronic Fatigue Clinic for a three-year history of exercise-induced asthma, progressive fatigue, and severe rhabdomyolysis. During episodes of rhabdomyolysis, usually triggered by physical exertion, his creatine kinase levels reached as high as 5956 U/L (reference range, 39-308 U/L). No etiology of the rhabdomyolysis was identified despite extensive metabolic testing; his muscle biopsy was normal, as were the plasma acylcarnitine profile and mitochondrial gene mutations. He reported progressive fatigue, post-exertional malaise (PEM), and experienced a severe decline in physical capability. Before the onset of symptoms, he was an active soccer player and good student, but at the time of consultation, he could only attend school every other day. He described gluten and dairy intolerance, heat intolerance, red cheeks since infancy, and keratosis pilaris. His mother had a history of angioedema since birth, with severe hives in reaction to multiple foods and direct sun exposure. Figure 1 illustrates the degree of facial erythema.

He satisfied the 2015 Institute of Medicine diagnostic criteria for myalgic encephalomyelitis/chronic fatigue syndrome (ME/CFS) [18] and criteria for neurally mediated hypotension (NMH) [15]. He also satisfied the Consensus-2 criteria for MCAS [11]. His serum tryptase levels were 1.3 and 1.9 mcg/L (reference range for this laboratory, < 11 mcg/L) on different occasions. A chromogranin A level was 34 ng/mL (reference range, < 93 mcg/mL). He described his wellness score as 20/100, with a score of 100 meaning optimal health.

Without changes in other medications, he began oral cromolyn, but did not see any improvement at the usual dose of 800 mg daily. As the dose was increased to 1200 mg daily (12 vials), he was able to sit upright all day, as opposed to previously always having to spend the day reclined. Once he increased to 1600 mg daily, he was able to tolerate trips out of the house, sitting in doctors’ offices (instead of lying down on the examination table), and did not develop post-exertional malaise after appointments. After a month at this dosage, with other medications unchanged, he continued to improve. He was now able to drive two hours or more without getting lightheaded, whereas previously he was only able to drive a mile before the lightheadedness became too severe. He no longer needed breaks to lie down during the day. Before the cromolyn, he was only upright 10-20 minutes daily, but after, he attended a soccer game that required walking two miles. He was able to go bowling with his friends, which was unthinkable 2-3 months prior. He estimated a wellness score of 70/100 seven months after beginning cromolyn. 

At a following appointment a few months later, there was a manufacturing shortage of oral cromolyn, which forced him to ration his cromolyn transiently. He felt substantially worse on lower doses of 400-800 mg daily; he described the effect as a ‘night and day’ difference, thereby confirming the utility of the medication for his MCAS.

A few months after the shortage, he tested positive for SARS-CoV-2 and did not recover well, which is a common pattern seen in ME/CFS patients and in individuals whose MCAS is inadequately treated at the time of infection [19-21]. To help return him to his baseline, we increased the oral cromolyn dose to 2000 mg daily. He was able to stay upright again, was much less lightheaded, and was able to drive again. He has been on his dosage for over a year with no side effects. Other medications added for control of MCAS after his cromolyn dose had been optimized had a minimal effect on overall function. These included fexofenadine 180 mg daily, famotidine 40 mg twice daily, montelukast 10 mg nightly, quercetin 1000 mg twice daily, and doxepin 10 mg nightly.

Case 2

A 37-year-old female in our clinic was diagnosed with ME/CFS, postural tachycardia syndrome (POTS), asthma, irritable bowel syndrome (IBS), neurogenic thoracic outlet syndrome (nTOS), constant daily headache, hypermobile Ehlers-Danlos syndrome (hEDS), hyperhidrosis, anxiety, and depression. Prominent mast cell activation symptoms included marked facial flushing, generalized skin erythema, recurring rashes affecting the upper chest and face, nasal congestion, as well as periorbital and facial swelling. Her wellness score at the initial evaluation was 26/100, at which time she was already being treated with cetirizine 10 mg daily and famotidine 40 mg twice daily. 

To improve headaches, nasal congestion, sinus inflammation, and periorbital swelling, she began taking Nasalcrom® at a dose of two sprays to each nostril twice daily. After a month, she reported 30% less skin erythema, lessened anxiety and depression, improved daily headache, resolved diarrhea, and improved brain fog. Due to the global response to the treatment, she then increased the Nasalcrom® dose to two sprays in each nostril four times daily. This continued to help her headaches, brain fog (specifically word finding), and gastrointestinal symptoms.

The positive response to the Nasalcrom® led us to prescribe oral cromolyn for the remaining MCAS symptoms she was experiencing. She started oral cromolyn at 400 mg daily, and did not see any difference until week two, when she increased the dose to eight vials daily, which improved her facial and periorbital swelling. After forgetting one day’s dose, she had severe bowel cramps and diarrhea.

She then added the eye drop form of cromolyn, Opticrom®, at a rate of one drop per eye four times a day. This helped substantially for the “gritty” feeling in her eyes and her conjunctival erythema. She eventually increased the dose to 2 drops per eye four times daily, which completely resolved eyelid swelling and cheek rashes.

At the following appointment, she reported that putting one vial of oral cromolyn in her nasal rinse once a day proved more effective than any other treatment for her facial swelling and sinus pressure. We recommended using the oral cromolyn as a topical treatment for exterior vaginal irritation, as has been reported by Afrin and colleagues [22]. She described an immediate effect of 65% improvement with a one-vial application. To maximize her improvements from cromolyn, she increased the oral dose to 1600 mg daily, at which point she reported that all her MCAS symptoms were stable. After eight months on these various forms of cromolyn, she reported a wellness score of 61/100. She was able to go from one day of activity weekly to working 5-7 days per week with no post-exertional increases in fatigue or pain. Laboratory testing while on cromolyn showed a serum tryptase level of 3.0 mcg/L (reference range 2.2-13.2 mcg/L), histamine 0.26 ng/mL (reference range, < 1.00 ng/mL), and chromogranin A 32.7 ng/mL (reference range 0-101.8 ng/mL).

Case 3

A 25-year-old male in our clinic was diagnosed with ME/CFS, POTS, chronic daily headaches, cranio-cervical instability (CCI), and nTOS. Symptoms and signs consistent with mast cell activation included facial flushing, scattered and recurrent rashes, erythema of the ears and hands, pruritus, and recurrent urticaria on the face and neck. His plasma histamine level was elevated to 12.6 ng/mL (reference range ≤ 1.8 ng/mL), with tryptase levels of 2.8-3.0 mcg/L (reference range < 11 mcg/L) and a chromogranin A of 164 ng/mL (reference range < 311 ng/mL). Loratadine 10 mg daily and cetirizine 10 mg nightly were associated with a modest improvement in energy; montelukast 10 mg nightly and famotidine 40 mg twice daily provided partial relief of facial flushing. Oral cromolyn at 800 mg daily was associated with significant reductions in his facial flushing, cutaneous erythema, rashes, hives, and itching. At a dose of 1600 mg daily, his facial flushing, rashes, and hives had completely resolved. After years of constant fatigue and minimal activity, he was able to resume his hobby of swimming. His wellness score had risen from 30/100 before the initiation of cromolyn to the 70-80/100 range on treatment for 12 months. He feels that his MCAS is completely stable.

Case 4

A 19-year-old female in our clinic was diagnosed with ME/CFS, POTS, hEDS, asthma, and allergic rhinitis. Symptoms and signs consistent with MCAS included fatigue associated with widespread cutaneous erythema, facial flushing, and recurrent urticaria (which was controlled with levocetirizine 5 mg daily and hydroxyzine 10 mg twice daily). She met the Consensus-2 diagnostic criteria for MCAS. Adding famotidine 40 mg twice daily provided only modest benefits. She noticed improvement in her orthostatic intolerance, fatigue, and brain fog, beginning at an oral cromolyn dose of 400 mg daily. At 800 mg per day, she noticed a decrease in cardiac awareness and improved stamina. At 1200 mg per day, she reported improved temperature regulation, as well as reduced flushing and lightheadedness. Because she still reported rashes in the shower, we increased the dose to 1600 mg per day, at which point her rashes, hives, and headaches all completely resolved. She also described improvement in coordination and fine motor skills, which we attributed to improved muscle strength. With the addition of cromolyn, her wellness score increased from 60/100 to 86/100. Laboratory studies obtained while on the various treatments for MCAS showed a tryptase level of 1.8 (reference range < 11.0 mcg/L), histamine < 1.5 (reference range ≤ 1.8 ng/mL), and chromogranin A level of 88 (reference range < 311 ng/mL).

Case 5 

A 41-year-old female with a prior history of asthma, chronic rhinitis, and Raynaud’s syndrome had been active, enjoying running and regular exercise, averaging about 10,000 steps daily. Her asthma and allergies had been treated with fluticasone nasal spray and fluticasone via inhalation, loratadine 20 mg twice daily, and montelukast 10 mg nightly. Four weeks after a probable SARS-CoV-2 infection in January 2021she developed profound fatigue, orthostatic intolerance, cognitive fogginess, and post-exertional malaise after minimal physical activity. She was primarily housebound, requiring a motorized wheelchair for activity outside the house. She ultimately satisfied diagnostic criteria for ME/CFS, MCAS, and Long COVID. Her serum tryptase level was 4.8 ng/mL (reference range 2.2-13.2 mcg/L), histamine 0.29 (reference range < 1.00 ng/mL), and chromogranin A level was < 20 (reference range 0-101.8 ng/mL). We began her on Nasalcrom® to treat chronic rhinitis and famotidine 20 mg twice daily to treat indigestion, diarrhea, bloating, and abdominal cramps. Although sinus inflammation was reduced, she still reported gastrointestinal symptoms. At this point, we prescribed oral cromolyn. She first reported symptom alleviation at 800 mg per day, with improvements not only in the gastrointestinal symptoms, but also in fatigue and brain fog. She reported needing to take fewer naps and was able to be more present with her family in the evenings. She tolerated more steps per day, going from 2000 steps per day, which provoked PEM, to 3500 steps per day without increased symptoms. When she increased the dose to 1600 mg daily, she had further improvements in all the symptoms listed above. A month later, however, there was a cromolyn manufacturing shortage, leaving her with no cromolyn for a few days. She immediately noted a significant decline in her wellness, characterized by an inability to care for her children. Once the medication was available again, the 1600 mg daily dose did not return her to the previous baseline, so we increased the dose to 2400 mg daily. This increase once again resulted in the alleviation of gastrointestinal symptoms, tolerance of four hours a day with her feet on the ground, 3000 steps daily without PEM, and reduced need for naps throughout the day. She has been on the 2400 mg/day dose for two years now, with no side effects and continuous benefit.

## Discussion

The cases we have described all benefited from a dose of cromolyn that is higher than usually recommended in the published literature for MCAS, but well within the safe dosage ranges of the original FDA application. Doses of 1600-2400 mg per day were well tolerated in patients for whom the 800 mg daily dose was ineffective. This draws attention to the option of further dose escalation in MCAS patients who have not responded entirely at the 800 mg/day dose. The recommended dose of oral cromolyn for mast cell dysfunction varies, with a typical suggested amount of 800 mg daily, but the dose can be increased up to 40mg/kg per day [12]. At an average weight of 60 kg, the maximum dose approved would be 2400 mg per day. All patients described in this case series weighed at least 60 kg, and their cromolyn doses were therefore under the 40 mg/kg/day maximum. Consistent with the doses used in our patients, the off-label uses for food allergy or irritable bowel syndrome recommend an upper limit of 1600 mg per day (400 mg 4x a day) [1,23].

A second practical observation is that the continuous oral dosing regimen was not only well tolerated, but it may also have the advantage of addressing mast cell triggers that are unrelated to food and mealtimes (e.g., pollen, mold, environmental exposures). Oral cromolyn sodium is poorly absorbed, with a bioavailability of 0.5-2%, where the rest of the unabsorbed active medication (98%) is excreted [1,23]. At a theoretical level, these drug kinetics would seem to favor gradual dosing throughout the day, rather than boluses of higher doses. More formal studies will be able to assess whether the continuous oral dosing regimen is as effective as four discrete doses per day and promotes improved adherence, as was reported by our patients.

Cromolyn has a variable onset of initial action, which can range from one day to six weeks, but the initial effect on the mast cells lasts for around six hours per dose. Oral cromolyn is packaged in ampules protected from light, because exposure reduces its half-life to 80-90 minutes [1]. A classic approach to treating MCAS begins with the introduction of antihistamines, which reduce the action of histamine after it has been released by mast cells. This treatment is not always completely effective in eliminating MCAS symptoms. Cromolyn inhibits mast cell degranulation, reducing the release of histamine and other mediators from mast cells. Cromolyn sodium has been shown to improve flushing, headaches, diarrhea, vomiting, nausea, urticaria, abdominal pain, and itching. It is currently FDA approved for prophylaxis of mild to moderate bronchial asthma, allergic rhinitis, mast cell dysfunction, mastocytosis, allergic eye conditions (conjunctivitis, keratitis, etc.), and nasal allergies. Off-label use of cromolyn has also shown efficacy in managing allergic reactions to foods, inflammatory bowel disease, vaginitis, and superior limbic keratoconjunctivitis [22].

Cromolyn has a low prevalence of adverse reactions or side effects. The most common reaction to topical use is irritation at the site of administration. The most common reactions to the oral form are headaches, diarrhea, nausea, abdominal pain, rash, and irritability, although many cases are difficult to confidently attribute to the cromolyn. These reactions were usually transient and might be attributed to the patients’ comorbid conditions [1]. Another potential source of reactions to oral cromolyn when packaged in plastic vials is contamination of the liquid medication by micro-particulate plastic dust remaining on the surface of the vial during production, or off-gassing from insufficiently aged plastic. Trials of liquid formulations in different containers or in a compounded form would be reasonable to consider in those who do react to the liquid form of the drug. Due to its poor absorption rate, there is a low toxicity, and the only restriction in prescribing cromolyn is in children younger than two years old [12]. This restriction is not based on evidence of adverse reactions, but simply due to insufficient research. 

Several aspects of the clinical presentations of the patients we report warrant further comment. First, the clinical features of mast cell activation syndrome have a substantial overlap with ME/CFS and Long COVID, making it challenging to isolate the independent contributions of each disorder to symptoms. However, the improvements noted after the initiation of cromolyn therapy in the patients we report are all consistent with effects of mast cell activation, most notably in cutaneous erythema, urticaria, and flushing. The improvements in systemic symptoms of lightheadedness, fatigue, and cognitive dysfunction are also understandable as consequences of improvements in orthostatic intolerance, consistent with reports of MCAS associated with POTS [24]. Although cromolyn is poorly absorbed and is often said to be active only at the sites of local application, it was associated with improvements in symptoms and systems distant from the site of direct application of the drug. Because mediators released from activated mast cells can have a range of effects on other tissues, reductions in mast cell degranulation and mediator release due to treatment with cromolyn would be expected to lead to improvements in delivery of those mediators to distant cells, tissues, organs, and systems. Rohrhofer and colleagues have estimated that up to 25.3% of ME/CFS patients meet criteria for MCAS [25]. Together with the substantial overlap in symptoms between the two conditions, these data would suggest that ascertainment for co-morbid MCAS would be important in the evaluation of those with ME/CFS because of the potential for symptomatic improvement with relatively benign medications directed at mast cell activation.

Second, the improvements we report have implications for those being evaluated for other conditions, ranging from dry eyes (Case 2) to Long COVID (Cases 1 and 5). Several groups have drawn attention to the potential for chronic symptoms to worsen after SARS-CoV-2 illness, especially when MCAS is not well controlled at the time of infection, as has been noted by several authors [19-21]. Case 2 had irritation of the eyes that was diagnosed as “dry eyes," improving after Opticrom® treatment. This raises the question of whether mast cell activation might be a cause of other forms of eye irritation that are misclassified as dry eye syndromes, especially in those with intact tear production.

Limitations: This was not a random sample of patients or a consecutive series, so our results cannot be considered representative of the overall response to this continuous oral dosing regimen of cromolyn. Our goal was simply to draw attention to the need to exceed the frequently recommended 800 mg/day maximum dose to reach optimal efficacy. We have not recorded the optimal dose for all patients seen in our clinic, nor have we collected data on the proportion of patients who appear to benefit from cromolyn at any dose, but these would be worthwhile objectives of future studies. We did not formally study compliance with the continuous oral cromolyn regimen and cannot be sure about how evenly patients distributed the dose throughout the day. In order to better assess whether gradual administration is more effective, we would need an extended-release preparation, but we are unaware of such a product.

We cannot be sure whether our continuous oral regimen maintains the drug’s stability over the day’s administration; however, the improvements noted would suggest cromolyn does remain stable for approximately 12 hours while in an opaque water bottle. It is also critical for cromolyn’s stability that the pH of the water remains between 2and 7, as alkaline water can initiate alkaline hydrolysis. If higher doses prove to be more effective, it would be more convenient to package the medication in higher concentrations per vial, which would decrease the time required to prepare the daily dose (potentially improving compliance). 

Alternative explanations for the improvements we report could include increased attention from specialist physicians or natural fluctuation in MCAS symptoms (although the improvements in all cases were prompt and in close temporal association with the changes in cromolyn dosing), or a placebo effect, acknowledging that placebo effects are not seen with increased frequency in ME/CFS [26].

Future studies of the continuous oral dosing regimen would benefit from consistent baseline mast cell mediator measurement, consistent measurement at standardized intervals of changes in symptoms and function using a wider range of survey instruments, randomization to either four discrete doses daily or the continuous oral daily regimen, and better measures of compliance. Given the six-hour duration of action of a dose of cromolyn, such randomized trials could help determine whether improved responses are due to the mode of administration (bolus versus continuous) or the higher dose alone. We chose to increase the cromolyn dose at weekly intervals, but evaluation of longer periods of time at each dose would be of interest. Randomized trials could also examine the cost-benefit ratio of the high dose versus the lower dose in lowering the number of medical appointments, emergency room visits, or hospitalizations. Pharmacokinetic studies are needed to determine whether dose escalation of cromolyn leads to non-linear pharmacokinetics, potentially resulting in higher serum levels and unexpected toxicity. While we did not see evidence of toxicity, the pharmacokinetics of cromolyn at different doses would also be a worthwhile focus of future studies.

## Conclusions

This retrospective case series suggests that using a higher-dose of oral cromolyn (1600-2400 mg/day), administered via continuous oral dosing during the day, may benefit selected ME/CFS patients with MCAS who have an inadequate response to standard 800 mg/day dosing. The continuous administration approach requires prospective validation comparing adherence and efficacy to conventional four-dose daily regimens. Controlled trials are needed to determine optimal dosing, strategies, and identify patient characteristics that predict a response to dose escalation. Administration of cromolyn using a continuous oral regimen has some theoretical advantages over four times a day. This retrospective case series suggests that using a higher-dose of oral cromolyn (1600-2400 mg/day), administered via continuous oral dosing during the day, may benefit selected ME/CFS patients with MCAS who have an inadequate response to standard 800 mg/day dosing. The continuous administration approach requires prospective validation comparing adherence and efficacy to conventional 4-dose daily regimens. Controlled trials are needed to determine optimal dosing strategies and to identify patient characteristics that predict a response to dose escalation. Administration of cromolyn using a continuous oral regimen has some theoretical advantages over four separate doses per day, although further study is needed.
